# Prognostic Role of Tissue Iron Deficiency Measured by sTfR Levels in Heart Failure Patients without Systemic Iron Deficiency or Anemia

**DOI:** 10.3390/jcm13164742

**Published:** 2024-08-13

**Authors:** Raúl Ramos-Polo, Maria del Mar Ras-Jiménez, Josep Francesch Manzano, Silvia Jovells-Vaqué, Herminio Morillas Climent, Alexandra Pons-Riverola, Sergi Yun Viladomat, Pedro Moliner Borja, Carles Diez-Lopez, José González-Costello, Elena Garcia-Romero, Lorena Herrador, Fernando de Frutos Seminario, Cristina Enjuanes Grau, Marta Tajes Orduña, Josep Comin-Colet

**Affiliations:** 1 Bio-Heart Cardiovascular Diseases Research Group, Bellvitge Biomedical Research Institute (IDIBELL), L’Hospitalet de Llobregat, 08907 Barcelona, Spain; 2 Community Heart Failure Program, Cardiology Department, Bellvitge University Hospital, L’Hospitalet de Llobregat, 08907 Barcelona, Spain; 3 Cardiology Department, Bellvitge University Hospital, L’Hospitalet de Llobregat, 08907 Barcelona, Spain; 4 Department of Internal Medicine, Bellvitge University Hospital, L’Hospitalet de Llobregat, 08907 Barcelona, Spain; 5 Centro de Investigación Biomédica en Red de Enfermedades Cardiovasculares (CIBERCV), 28029 Madrid, Spain; 6 Advanced Heart Failure and Heart Transplant Program, Cardiology Department, Bellvitge University Hospital, L’Hospitalet de Llobregat, 08907 Barcelona, Spain; 7 Department of Clinical Sciences, School of Medicine, University of Barcelona, 08007 Barcelona, Spain

**Keywords:** chronic heart failure, comorbidities, iron deficiency, biomarkers, clinical outcomes

## Abstract

**Background**. Iron deficiency (ID) is a significant, high-prevalence comorbidity in chronic heart failure (HF) that represents an independent predictor of a worse prognosis. However, a clear-cut diagnosis of ID in HF patients is not assured. The soluble transferrin receptor (sTfR) is a marker that reflects tissue-level iron demand and may be an early marker of ID. However, the impact of sTfR levels on clinical outcomes in non-anemic HF patients with a normal systemic iron status has never been evaluated. **Methods**. This is a post hoc analysis of an observational, prospective cohort study of 1236 patients with chronic HF of which only those with normal hemoglobin levels and a normal systemic iron status were studied. The final cohort consisted of 215 patients. Tissue ID was defined as levels of sTfR > 75th percentile (1.65 mg/L). Our aim was to describe the association between sTfR and clinical outcomes (all-cause death and HF hospitalization) and to explore its association with a wide array of serum biomarkers. **Results**. The sTfR level (HR 1.48, 95% CI 1.13–1.96, *p* = 0.005) and tissue ID (HR 2.14, 95% CI 1.22–3.75, *p* = 0.008) was associated with all-cause death. However, we found no association between sTfR levels and the risk of HF hospitalization. Furthermore, high sTfR levels were associated with a worse biomarker profile indicating myocardial damage (troponin and NT-proBNP), systemic inflammation (CRP and albumin), and impaired erythropoiesis (erythropoietin). **Conclusions.** In this cohort, the presence of tissue ID defined by sTfR levels is an independent factor for all-cause death in patients with normal systemic iron parameters.

## 1. Introduction

Heart failure (HF) is a very prevalent disease that has an enormous clinical impact. It affects patient quality of life (QoL) and causes cardiovascular and non-cardiovascular deaths. Moreover, it entails a high healthcare expense due to diagnostic procedures, hospitalization, and treatments [1]. Iron deficiency (ID) is an important comorbidity in HF that has a high prevalence rate of around 40–70% [2,3]. Regardless of the presence of anemia, it represents an independent predictor of worse QoL, functional capacity, and prognosis in patients with chronic HF [4,5,6]. Several studies have shown that intravenous iron replacement improves functional capacity and QoL in patients with HF [7,8]. Additionally, it may reduce the risk of recurrent cardiovascular hospitalizations [9]. Therefore, the correct identification of HF patients that rightly present with ID is essential since it modifies their clinical management and can decisively affect their prognosis.

Nevertheless, a clear-cut diagnosis of ID in patients is not assured. Although the gold standard is the assessment of iron stores directly in bone marrow, its invasiveness limits its clinical application. Thus, ID definition in the context of HF is based on ferritin < 100 μg/L or ferritin between 100 and 300 μg/L with transferrin saturation (TSAT) < 20% [3]. However, the accuracy of these criteria is in the spotlight. Firstly, ferritin might be modified by many other conditions other than ID [10,11]. In addition, most experts suggest that systemic iron depletion in HF develops as a continuum, starting from a normal iron status and going on to overt systemic ID [12,13,14]. The first stage of this spectrum would be a mild functional ID at the tissue level that has no impact on iron storage and transport compartments. In this regard, it has been suggested that the soluble transferrin receptor (sTfR) better reflects iron demand at the tissue level at an exceedingly early stage. Previous works have suggested that sTfR might be the best candidate as a screening tool for ID in HF patients [15] while also showing associations with clinical outcomes, functional capacity, and QoL [15,16]. 

However, those studies were made in cohorts with a great proportion of patients with anemia and overt systemic ID in accordance with the current definition of ID. Therefore, the capacity of sTfR to predict outcomes in HF patients without systemic ID or anemia has never been explored. 

Given the above-mentioned limitations, our study aims to determine whether tissue ID (defined by sTfR levels) can predict clinical outcomes in HF patients with a normal systemic iron status. In addition, our group also explored the relationship of sTfR with a broad panel of biomarkers that provide indicative data on myocardial damage, renin–angiotensin–aldosterone system (RAAS) activation, erythropoiesis, and inflammatory status.

## 2. Materials and Methods

Patient selection. This research is based on the DAMOCLES cohort, consisting of 1236 consecutive heart failure patients, enrolled between January 2004 and January 2013 in a single-center, prospective observational study [16]. Inclusion required a heart failure diagnosis based on the European Society of Cardiology criteria and a history of at least one recent acute heart failure episode necessitating intravenous diuretic treatment. Patients were excluded if they had significant primary valvular disease, clinical signs of fluid overload, pericardial disease, restrictive cardiomyopathy, hypertrophic cardiomyopathy, hemoglobin (Hb) levels below 8.5 g/dL, active cancer, or chronic liver disease.

For this specific analysis, we explored patients who had a thorough iron status assessment, including sTfR levels, normal hemoglobin (≥12 g/dL), and normal systemic iron parameters (serum iron > 33 µg/dL, ferritin > 100 μg/L, and transferrin saturation > 20%). This subgroup comprised 215 patients.

Ethical committee and data availability. The study was approved by the local ethics committee for clinical research and was conducted in accordance with the principles of the Declaration of Helsinki. All patients gave written informed consent before study entry. The data that support the findings of this study are available on request from the corresponding author.

Parameters collected. At study entry, all participants underwent a complete baseline evaluation. Demographic characteristics, clinical and disease-related factors like the New York Heart Association (NYHA) functional class and co-morbidities, laboratory tests, medical treatments, and the most recent left ventricle ejection fraction (LVEF) were collected. The medical history was the main source of information. Information regarding hospital admissions and survival was obtained from the HF clinical database or from the hospital system. 

Outcomes. The aim of the present study was to describe the association between sTfR levels and clinical outcomes. On this matter, the primary and secondary outcomes were all-cause death and HF hospitalization, respectively. Moreover, we also investigated the association between sTfR and a wide array of serum biomarkers in non-anemic patients with HF and normal systemic iron parameters. The relationship between cardiac biomarkers (NTproBNP, troponin), neurohormonal biomarkers related to the RAAS (aldosterone, angiotensin convertor enzyme (ACE) activity, renin activity), cellular response to hypoxia (erythropoietin), and biomarkers of inflammatory status (albumin, C-reactive protein (CRP)) were also explored.

Tissue ID definition. The sTfR levels were determined using the Beckman Coulter enzyme immunoassay. While higher sTfR levels indicate a greater iron demand, there is no standardized and validated cutoff value for sTfR that defines tissue ID. In this study, tissue ID was characterized by sTfR levels exceeding the 75th percentile, equating to 1.65 milligrams per liter (mg/L).

Statistical analysis. A descriptive analysis was carried out using baseline data from the DAMOCLES cohort. Demographic and clinical characteristics, as well as laboratory test results, were summarized with basic descriptive statistics. The cohort was divided depending on the presence or absence of tissue ID as defined by the sTfR level.

Categorical variables were presented as numbers and percentages, while continuous variables were summarized using mean (standard deviation) or median (interquartile range), depending on their distribution. Comparisons across strata were conducted using χ^2^ tests, Student’s *t*-tests, and non-parametric tests as appropriate.

We utilized unadjusted generalized additive models (GAMs) to examine both parametric and non-parametric relationships between sTfR levels (1 mg/L increase) and the biomarkers. These findings were confirmed in a multivariate adjusted GAM model. To further investigate the associations between sTfR levels, tissue ID, and the study biomarkers, we developed univariate and multivariate linear regression models. All multivariable models were adjusted for age, sex, and LVEF. Extreme sTfR values (>3 mg/dL) were excluded for this analysis.

Finally, we constructed multivariate Cox proportional hazards models to examine the associations between tissue ID and clinical outcomes. Additionally, we used GAM to explore the parametric and non-parametric relationships between sTfR levels and the estimated β risk of primary and secondary outcomes. In this case, multivariate models were adjusted for age, sex, and prognostic factors like the LVEF, NYHA, ischemic etiology, comorbidities (diabetes mellitus, hypertension, chronic kidney disease and obesity), biomarkers (NTproBNP and albumin levels), neuro-hormonal treatment (beta-blockers, angiotensin convertor enzyme inhibitors (ACEI), angiotensin receptor blockers (ARB), and mineralocorticoids receptor antagonists (MRA)).

For the multivariable linear regression models, backward conditional stepwise methods were utilized. Afterward, a collinearity assessment was carried out to verify that all variables in the final model had a tolerance level greater than 0.3, thereby ruling out significant collinearity. Finally, all the variables included in the model had a tolerance between 0.95 and 1.00.

Statistical analyses and confidence intervals (CI) were calculated with a Type I error rate set at 5%, without adjustments for multiple comparisons. *p*-values less than 0.05 were deemed statistically significant. The analyses were conducted using SPSS software (version 22.0; IBM, Armonk, NY, USA) and R software (version 4.2.1; R Foundation for Statistical Computing, Vienna, Austria).

## 3. Results

**Figure 1:**
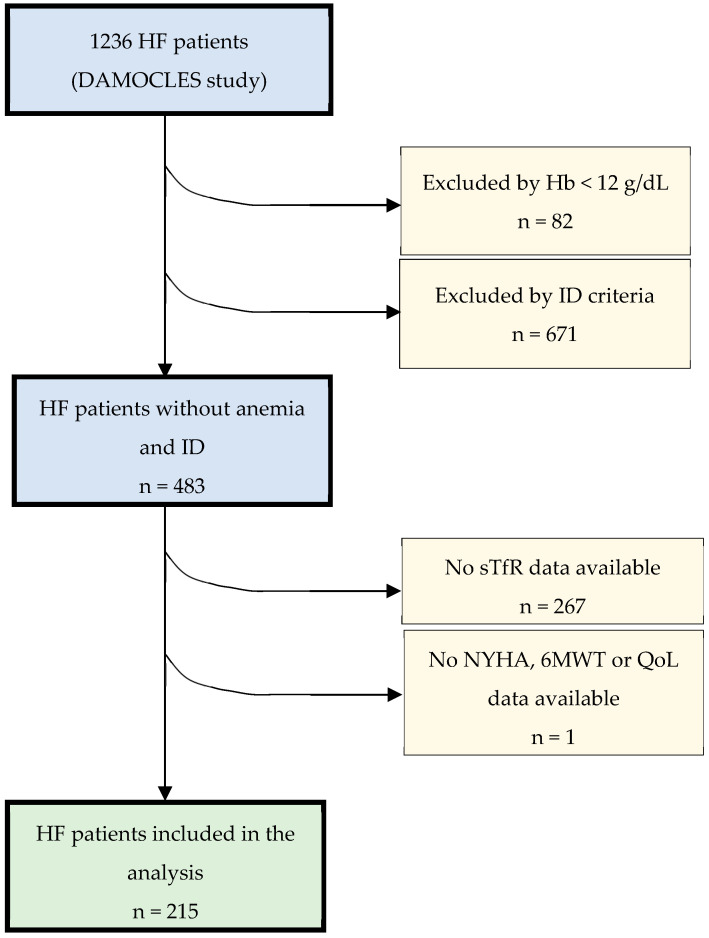
Flowchart and exclusion criteria.

The DAMOCLES study enrolled 1236 patients with HF and the entire LVEF spectrum. For the present study, only those patients without anemia and ID were studied. The cohort finally included 215 patients (Figure 1).

### 3.1. Baseline Patient Characteristics

The baseline characteristics of the study sample, both overall and according to tissue ID status (tissue ID ≥ 1.65 mg/L, defined as levels of sTfR > 75th percentile) are listed in Table 1. Tissue ID was present in 54 patients (25%). The mean age was 70 ± 12 years, 62 (29%) were women and the mean LVEF was 43 ± 15%. There were no differences between groups in terms of age, LVEF, or the etiology of HF (all *p*-value > 0.05). The mean sTfR values were 1.42 ± 0.66 mg/L. The mean hemoglobin levels were 14.1 g/dL and were similar between groups (14.2 vs. 14.0 g/dL, *p* = 0.447).

Interestingly, women were heterogeneously distributed (24 vs. 43% favoring tissue ID group, *p* = 0.010). Furthermore, the tissue ID group showed a higher heart rate (72 vs. 78 bpm, *p* = 0.010), poorer functional class (NYHA III–IV 24% vs. 35%, *p* = 0.020), and covered a shorter distance in the 6MWT (314 vs. 206 m, *p* < 0.001). The mean sTfR values were 1.42 ± 0.66 mg/L and the mean hemoglobin levels were 14.1 g/dL. The tissue ID group had slightly worse renal function (eGFR 70 vs. 60 mL/min/Kg, *p* = 0.018)

Both groups were similarly treated with beta-blockers, ACEi or ARBs, and MRA (86%, 89% and 42%, respectively), all *p*-values > 0.05. The proportion of patients with atrial fibrillation was higher in the tissue ID group (59% vs. 37%, *p* = 0.005). Accordingly, more patients in the tissue ID group were treated with anticoagulant therapy compared with the no tissue ID group (47% vs. 65%, *p* = 0.020) while no differences were seen regarding antiplatelet treatment.

### 3.2. sTfR Association with Clinical Outcomes

During the 5-year follow-up, 25.6% of patients required hospitalization for acute HF. The all-cause death rate at 5 years was 27.9%.

**Figure 2:**
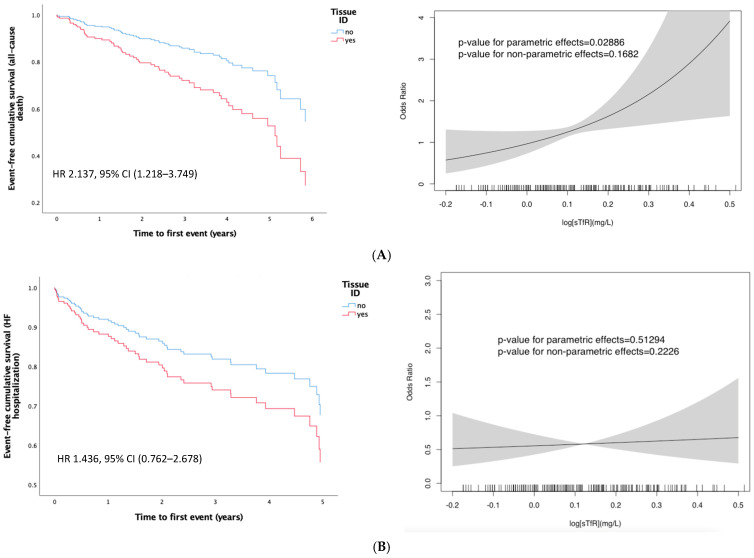
Multivariate Cox proportional hazards showing event-free cumulative survival for clinical outcomes (all-cause death and HF hospitalization) according to presence of tissue ID in the cohort of non-anemic patients with HF and normal systemic iron parameters. Multivariate Generalized Additive Models (GAM) exploring the associations between sTfR levels and clinical outcomes (all-cause death and HF hospitalization). (**A**) all-cause death. (**B**) HF hospitalization.

Multivariate models (Table 2) were adjusted for age, sex, and prognostic factors. Both sTfR levels (HR 1.484, 95% CI 1.125–1.958, *p* = 0.005) and tissue ID (HR 2.137, 95% CI 1.218-3.749, *p* = 0.008) were associated with all-cause death (Figure 2A). However, neither sTfR levels nor the presence of tissue ID were related with HF hospitalization (all *p*-values > 0.05) (Figure 2B).

Adjusted GAM (Figure 2A) was used to evaluate the interplay between sTfR levels and all-cause death revealed a direct and significant association between increased iron demand (higher levels of sTfR) and higher risk of mortality (*p*-value for parametric effects = 0.028). This association was not observed between sTfR levels and the HF hospitalization rate (Figure 2B).

### 3.3. sTfr Association with Cardiac, Hematinic, Inflammatory and RAAS Biomarkers

The overall laboratory values (hematinic, cardiac, RAAS activation, and inflammatory biomarkers) according to tissue iron status are shown in Table 3. Tissue ID patients had a higher median NTproBNP (1031 (496–2329) vs. 1768 (916–4130) pg/mL, *p* = 0.016), higher median erythropoietin (9 (6–16) vs. 11 (8–19) mUI/mL, *p* = 0.010) and lower mean albumin (4.1 ± 0.5 vs. 3.9 ± 0.7 g/dL, *p* = 0.024). There were no differences between the other biomarkers like the cardiac (troponin), hematinic (ferritin, TSAT and serum iron), RAAS activation (ACE activity, renin activity and aldosterone), and the inflammatory (CRP).

Unadjusted GAM (Figure S1) explored the interplay between sTfR levels and a wide array of biomarkers. A significant linear association was found between higher levels of sTfR (increased iron demand indicating tissue ID) and higher levels of NTproBNP (*p*-value for parametric effects 0.008), indicating cardiac damage and higher levels of erythropoietin (*p*-value for parametric effects 0.008), indicating tissue response to hypoxia. There were also higher levels of CRP (*p*-value for parametric effects < 0.001) combined with lower albumin levels, suggesting the presence of inflammatory status. A linear association between sTfR levels and troponin and RAAS activation was not observed (all *p*-values for parametric effects > 0.05).

The biomarker association with both the sTfR levels and tissue ID (sTfR > 75th percentile) were explored through regression models (Table 4). Higher sTfR levels, indicating increased iron demand, were associated with higher NTproBNP concentration (standardized β = 0.177, *p* = 0.009) and higher erythropoietin levels (standardized β = 0.180, *p* = 0.008) as well as inflammatory biomarkers including CRP (standardized β = 0.303, *p* < 0.001) and albumin (standardized β = −0.172, *p* = 0.012). These findings were confirmed in backwards conditional stepwise multivariate linear regression models (Table 4) and in multivariate GAM (parametric *p*-value < 0.05) (Table S1). All multivariable models were adjusted for age, sex, and LVEF. Once again, these models did not show an association between sTfR levels and troponin nor RAAS activation (all *p*-value for parametric effects > 0.05).

## 4. Discussion

**Figure 3:**
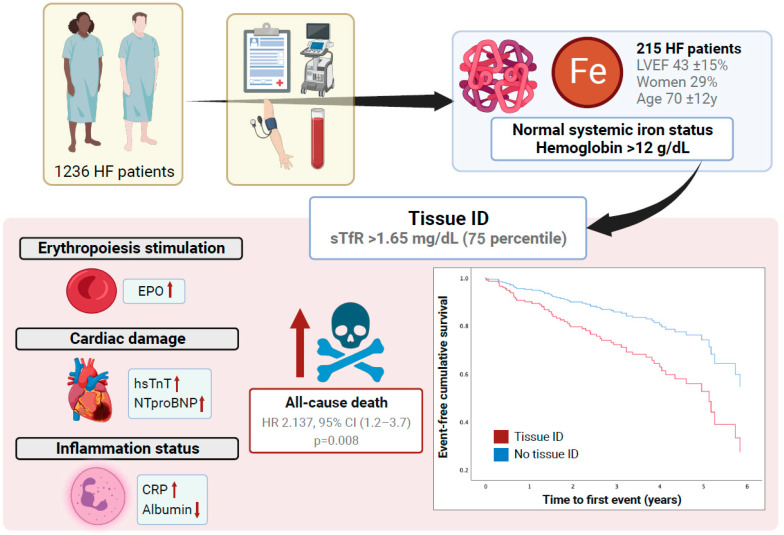
The presence of tissue ID (sTfR levels > 1.65 mg/dL) is an independent factor for all-cause death in patients with normal systemic iron parameters. High sTfR levels were associated with a worse biomarker profile indicating myocardial damage (troponin and NT-proBNP), systemic inflammation (CRP and albumin), and impaired erythropoiesis (erythropoietin).

In this study, we have shown that the presence of tissue ID (sTfR ≥ 1.65 mg/L) in patients with HF without anemia or systemic ID is an independent predictor for all-cause death. Both the presence of tissue ID and increased sTfR levels were associated with a worse biomarker profile suggestive of myocardial damage (higher NTproBNP levels), a pro-inflammatory state (suggested by higher CRP levels and lower serum albumin levels), and tissue hypoxia (higher erythropoietin levels). To our best knowledge, our research is the first to demonstrate that sTfR is a robust predictor of all-cause death in an HF patient population without anemia or systemic ID (Figure 3). In this instance, higher sTfR levels are associated with a worse biomarker profile, suggestive of myocardial damage, systemic inflammation, and tissue hypoxia.

This study is in line with previous work that has already proposed sTfR as a good predictor of mortality [15,17]. However, limitations such as small sample size, the low presence of women (less than 20%), the exclusion of patients with preserved LVEF, and the inclusion of patients with anemia and ID limit the extension of the results to other populations of patients with HF. The aim of this study was to complement previous research and provide information in patients without anemia and/or systemic ID. It explored the prognostic ability of sTfR in a broad-spectrum, real-world cohort of patients regardless of age and LVEF.

sTfR, which is expressed by almost all proliferating cells, is an established marker that provides useful information on cellular iron demands [18] that can provide necessary information to define the tissue iron status, especially given the limitations of the ID criteria (ferritin < 100 μg/L or ferritin between 100 and 300 μg/L and TSAT < 20%). Both inflammation and oxidative stress (quite common in the context of a chronic disease such as HF) can increase ferritin levels independently of iron levels [12]. On the other hand, decreased transferrin levels in a catabolic or malnutrition context may falsely elevate TSAT levels [12]. 

Furthermore, sTfR is a reliable parameter to define ID in bone marrow. Leszek et al. compared explanted failing hearts referred for heart transplantation to non-failing hearts in patients who died from head trauma and observed that sTfR is the only biomarker that correlated with myocardial iron levels [19], whereas serum levels of ferritin and TSAT were not associated with myocardial iron. Similarly, Sierpinski et al. described that sTfR had the best accuracy to predict ID in bone marrow in a population of patients with stable ischemic HF and an LVEF ≤ 45% [15].

As a prognostic tool, sTfR has been evaluated [15] in models adjusted for NTproBNP, hemoglobin, ferritin, and TSAT. The optimal cutoff point for predicting 3-year mortality was 1.41 mg/dL (80.1% vs. 62.8% 3-year survival). Likewise, high sTfR levels have also been associated with a poor prognosis in the acute HF setting [17]. It has also been identified as a determinant of submaximal exercise capacity independent of anemia [8,9]. Even in patients without ID or anemia, sTfR is strongly associated with an impaired submaximal exercise capacity and worse QoL [16]. Increased serum sTfR levels were also associated with a high prevalence of cardiovascular diseases [20] and has shown an association with higher blood pressure, HbA1c and glucose levels during oral glucose tolerance tests in populations with or without diabetes [21].

A great number of patients with HF receive intravenous iron therapy in real life, but more so when patients have a combination of anemia and ID than in cases of isolated ID as defined by the ESC guidelines [3]. However, the criteria used are derived from the inclusion criteria of first clinical trials [7] and extrapolation from patients with renal disease. Even though TSAT < 20% is associated with a higher risk of all-cause death, patients with a ferritin of <100 μg/L but with TSAT > 20% exhibit different clinical features and response to treatment [10]. The definition of ID needs to be refined to detect which patients really need iron replacement but currently do not receive it. Additionally, a determination must be made for the individuals for whom this treatment may be futile or even harmful.

Notably, our group has studied a novel scenario, which is HF patients without systemic ID and anemia. We observed an association between the sTfR levels and erythropoietin levels (hematopoietic pathway) as well as with the systemic biomarkers of myocardial damage (NTproBNP) and inflammation (CRP and albumin). That is a fact that underlines the role of iron in non-hematopoietic pathways. On the other hand, no association was demonstrated between sTfR levels and biomarkers, suggesting RAAS activation (renin, ACE, and aldosterone). The relevance of iron in non-hematopoietic pathways and its fundamental role in cellular metabolism justifies the clear association of tissue ID with all-cause death in a model adjusted for contrasted prognostic variables (including age, LVEF, comorbidities, NTproBNP, and neurohormonal treatment, among others). Finally, it is noteworthy that tissue ID was not associated with HF admissions. The sample size and the definition of HF hospitalization (which did not include decompensations treated in day hospital) might justify this lack of a relationship.

Our data strongly supports the use of sTfR for a more accurate and early definition of ID in HF patients. With a high sTfR level being related to deficient erythropoiesis in its initial stages, it suggests ID at functional protein levels. Remarkably, sTfR has demonstrated prognostic value for the prediction of death from any cause. sTfR could thereby enhance the performance of the standard ferritin and TSAT criteria given their limitations, particularly of isolated hypoferritinemia. Future research is needed to clarify the correlation of sTfR with systemic iron deficit, specifically with TSAT < 20%, to establish a standardized sTfR cutoff value. That research would also address the clinical outcomes derived from intravenous iron therapy in individuals with sTfR-defined tissue ID.

Some limitations to this study must be acknowledged. Firstly, this is a post hoc analysis. Therefore, the original study was not designed for the present end-point. Second, causality may not be inferred and all biases in relation to retrospective observational studies must be considered. Third, only all-cause death was encoded. With that in mind, no inference can be made regarding cardiovascular mortality. HF decompensations treated in outpatient hospital were not considered as HF admissions. It is a fact that may have attenuated the differences between groups. Fourth, neither angiotensin receptor–neprilysin inhibitors nor sodium-glucose cotransporter-2 inhibitors are included as neuro-hormonal because the date of the inclusion of patients in the DAMOCLES was prior to the introduction of these treatments into clinical practice. Fifth, there is no standardized cutoff value for sTfR which defines tissue ID. Hence, the results may undergo some variations based on the definition used. Finally, as this was a single-center study with a limited sample size, the conclusions cannot be applied to other HF populations. Randomized studies with a larger sample size are required to confirm the hypothesis and validate the results. 

## 5. Conclusions

In conclusion, our research demonstrates that higher sTfR levels are strongly associated with all-cause death in patients with HF and normal systemic iron parameters (without systemic ID). Furthermore, sTfR levels were associated with a panel of biomarkers, suggesting subclinical myocardial damage, tissue hypoxia, and inflammatory status. sTfR may be a good marker of impaired erythropoiesis and increased tissue-level iron demand even with normal ferritin and TSAT values.

## Figures and Tables

**Table 1:** Demographic and clinical characteristics of all patients included in this analysis, overall and according to tissue iron status (sTfR ≥ 1.65 mg/L indicating tissue ID).

	Whole Cohort (n = 215)	No Tissue ID (sTfR < 1.65 mg/L) (n = 161)	Tissue ID (sTfR ≥ 1.65 mg/L) (n = 54)	*p*-Value
**Demographics**				
Age, years	70 (12)	69 (12)	73 (12)	0.072
Sex (female), n (%)	62 (29%)	39 (24%)	23 (43%)	0.010
Systolic blood pressure, mmHg	125 (24)	126 (24)	123 (25)	0.366
Heart rate, bpm	73 (15)	72 (14)	78 (17)	0.010
NYHA Functional Class, n (%)				0.020
I	44 (21%)	39 (24%)	5 (9%)	
II	112 (53%)	82 (52%)	30 (57%)	
III	46 (22%)	33 (21%)	13 (24%)	
IV	11 (5%)	5 (3%)	6 (11%)	
6 min walking test, meters	287 (168)	314 (155)	206 (179)	<0.001
BMI, Kg/m^2^	28 (6)	28 (6)	28 (6)	0.849
HF hospitalization previous year, n (%)	172 (80%)	126 (79%)	46 (85%)	0.303
LVEF, %	43 (15)	43 (15)	43 (16)	0.942
**Comorbidities**				
Ischaemic etiology of HF, n (%)	64 (30%)	44 (27%)	20 (37%)	0.177
Hypertension, n (%)	156 (73%)	114 (71%)	42 (78%)	0.321
Diabetes Mellitus, n (%)	68 (32%)	54 (34%)	14 (26%)	0.298
Obesity, n (%)	58 (27%)	43 (27%)	15 (28%)	0.878
Previous MI, n (%)	35 (16%)	24 (15%)	11 (20%)	0.347
CKD, n (%)	90 (42%)	63 (40%)	27 (50%)	0.182
**Treatment**				
ACEI or ARBs, n (%)	185 (86%)	139 (86%)	46 (85%)	0.833
Beta-blockers, n (%)	191 (89%)	142 (88%)	49 (91%)	0.608
MRA, n (%)	90 (42%)	69 (43%)	21 (39%)	0.609
Diuretics, n (%)	195 (91%)	142 (88%)	53 (98%)	0.029
Antiplatelet therapy, n (%)	79 (37%)	64 (40%)	15 (28%)	0.114
Anticoagulant therapy, n (%)	110 (51%)	75 (47%)	35 (65%)	0.020
**Laboratory**				
Hemoglobin, g/dL	14.1 (1.4)	14.2 (1.3)	14.0 (1.5)	0.447
Creatinine, mg/dL	1.2 (0.4)	1.1 (0.3)	1.3 (0.5)	0.047
Estimated glomerular filtration rate, mL/min/kg	67 (26)	70 (26)	60 (25)	0.018
Serum proteins, g/dL	6.9 (0.7)	6.9 (0.7)	6.8 (0.7)	0.701
sTFR (mg/L)	1.42 (0.7)	1.15 (0.2)	2.25 (0.82)	0.000

NYHA: New York Heart Association. BMI: body mass index. HF: heart failure. LVEF: left ventricular ejection fraction. MI: myocardial infarction. CKD: chronic kidney disease, defined as estimated glomerular filtration (eGFR) date < 60 mL/min/1.73 m^2^. ACEi: angiotensin-converting enzyme inhibitors. ARBs: angiotensin receptor blockers. MRA: mineral corticoid receptor antagonists. Percentage may not sum 100% because of rounding.

**Table 2:** Multivariate (adjusted) Cox proportional hazards analyses exploring the effect on all-cause death and HF hospitalization of sTfR levels and tissue ID in the cohort of non-anemic patients with HF and normal systemic iron parameters (backward stepwise method).

All-Cause Death			
Measures of Tissue ID	HR	95% CI	*p*-Value
sTfR, 1 mg/L	1.484	1.125–1.958	0.005
sTfR > 75th percentile (1.63 mg/L)	2.137	1.218–3.749	0.008
**Heart Failure Hospitalization**			
**Measures of Tissue ID**	**HR**	**95% CI**	***p*-Value**
sTfR, 1 mg/L	1.241	0.876–1.759	0.225
sTfR > 75th percentile (1.63 mg/L)	1.436	0.762–2.678	0.260

**Table 3:** Overall laboratory values (hematinic, cardiac, renin–angiotensin–aldosterone system activation, and inflammatory biomarkers) according to tissue iron status (sTfR ≥ 1.65 mg/L indicating tissue ID).

	Whole Cohort (n = 215)	No Tissue ID (sTfR < 1.65 mg/L) (n = 161)	Tissue ID (sTfR ≥ 1.65 mg/L) (n = 54)	*p*-Value
Laboratory Values				
NT-proBNP, pg/mL (median, IQR)	1125 (587–2668)	1031 (496–2329)	1768 (916–4130)	0.016
Troponin, ng/mL (median, IQR)	0.010 (0.009–0.034)	0.010 (0.009–0.031)	0.012 (0.010–0.012)	0.190
Serum proteins, g/dL	6.9 (0.7)	6.9 (0.7)	6.8 (0.7)	0.701
Serum albumin, g/dL	4.0 (0.6)	4.1 (0.5)	3.9 (0.7)	0.024
Ferritin, ng/mL (median, IQR)	249 (154–432)	268 (166–440)	232 (130–296)	0.326
TSAT, %	30 (10)	30 (9)	29 (10)	0.233
Serum iron, ug/dL	102 (41)	103 (35)	98 (57)	0.509
Erythropoietin, mUI/mL (median, IQR)	10 (6–17)	9 (6–16)	11 (8–19)	0.010
ACE activity, U/L (median, IQR)	12 (7.2–21.5)	12 (6–19)	12 (11–24)	0.498
Plasmatic renin activity, ng/mL/h (median, IQR)	3.7 (1.2–15.7)	3.3 (1.2–22.1)	4.8 (1.4–11.5)	0.844
Aldosterone, pg/mL (median, IQR)	74.5 (33.0–148.3)	65 (32–156)	93 (44–93)	0.450
C-reactive protein, mg/dL (median, IQR)	0.42 (0.20–1.10)	0.40 (0.20–0.90)	0.72 (0.20–2.15)	0.429

TSAT: transferrin saturation. ACE: angiotensin-converting enzyme. IQR: interquartile range.

**Table 4:** Univariate and multivariate adjusted linear regression models exploring the effect on the biomarkers that indicate cardiac damage (troponin and NT-proBNP), renin–angiotensin–aldosterone system activation (aldosterone, serum ACE activity and plasma renin activity), inflammatory status (C-reactive protein), and cellular response to tissular hypoxia (endogenous erythropoietin) of sTfR and tissue ID in the cohort of non-anemic patients with HF and normal systemic iron parameters.

Cardiac Biomarkers					
Univariate Linear Regression Models			Multivariate Linear Regression Models		
Measures of Tissue ID	Standardized β Coefficient	*p*-Value	Standardized β Coefficient	*p*-Value	R Model
**Troponin**					
sTfR (1 mg/L)	0.136	0.131	0.088	0.333	0.041
sTfR > 75th percentile (1.65 mg/L)	0.039	0.664	0.014	0.104	0.030
**NTproBNP**					
sTfR (1 mg/L)	0.177	0.009	0.168	0.014	0.036
sTfR > 75th percentile (1.65 mg/L)	0.127	0.062	0.112	0.045	0.020
**Renin–Angiotensin–Aldosterone System Biomarkers**					
**Univariate Linear Regression Models**			**Multivariate Linear Regression Models**		
**Measures of Tissue ID**	**Standardized β Coefficient**	***p*-Value**	**Standardized β Coefficient**	***p*-Value**	**R Model**
**Aldosterone**					
sTfR (1 mg/L)	0.095	0.168	0.109	0.113	0.029
sTfR > 75th percentile (1.65 mg/L)	0.057	0.407	0.076	0.035	0.022
**Serum ACE Activity**					
sTfR (1 mg/L)	0.037	0.589	0.039	0.154	0.005
sTfR > 75th percentile (1.65 mg/L)	0.049	0.482	0.069	0.225	0.005
**Plasma Renin Activity**					
sTfR (1 mg/L)	−0.014	0.845	−0.015	0.421	0.003
sTfR > 75th percentile (1.65 mg/L)	0.026	0.705	0.009	0.784	−0.011
**Inflammation Biomarkers**					
**Univariate Linear Regression Models**			**Multivariate Linear Regression Models**		
**Measures of Tissue ID**	**Standardized β Coefficient**	***p*-Value**	**Standardized β Coefficient**	***p*-Value**	**R Model**
**C-reactive Protein**					
sTfR (1 mg/L)	0.303	<0.001	0.294	<0.001	0.097
sTfR > 75th percentile (1.65 mg/L)	0.189	0.006	0.174	0.005	0.041
**Albumin**					
sTfR (1 mg/L)	−0.172	0.012	−0.155	<0.001	0.068
sTfR > 75th percentile (1.65 mg/L)	−0.154	0.024	−0.131	<0.001	0.061
**Cellular Response to Hypoxia**					
**Univariate Linear Regression Models**			**Multivariate Linear Regression Models**		
**Measures of Tissue ID**	**Standardized β Coefficient**	***p*-Value**	**Standardized β Coefficient**	***p*-Value**	**R Model**
**Erythropoietin**					
sTfR (1 mg/L)	0.180	0.008	0.176	0.003	0.044
sTfR > 75th percentile (1.65 mg/L)	0.150	0.028	0.148	0.009	0.035

## Data Availability

Data is contained within the article or Supplementary Material.

## Supplementary Materials

The following supporting information can be downloaded at https://www.mdpi.com/article/10.3390/jcm13164742/s1, Figure S1: Boxplots (showing the mean and standard deviation) of biomarkers according to the presence (Tissue ID [+]) or absence (Tissue ID [−]) and Univariate Generalized Additive Models (GAM) exploring the associations between sTfR levels and the biomarkers that indicate cardiac damage (troponin and NT-proBNP), renin–angiotensin–aldosterone system activation (aldosterone, serum ACE activity and plasma renin activity), inflammatory status (C-reactive protein) and cellular response to tissular hypoxia (endogenous erythropoietin) tissue ID; Table S1: Sex, age and LVEF adjusted GAM to explore the parametric and non-parametric associations between sTfR and biomarkers indicating cardiac damage (troponin and NT-proBNP), renin–angiotensin–aldosterone system activation (aldosterone, serum ACE activity and plasma renin activity), inflammatory status (C-reactive protein) and cellular response to tissular hypoxia (endogenous erythropoietin).

## References

[1] Savarese G., Becher P.M., Lund L.H., Seferovic P., Rosano G.M., Coats A.J. (2023). Global burden of heart failure: A comprehensive and updated review of epidemiology. Cardiovasc. Res..

[2] Alnuwaysir R.I.S., Hoes M.F., van Veldhuisen D.J., van der Meer P., Beverborg N.G. (2021). Iron Deficiency in Heart Failure: Mechanisms and Pathophysiology. J. Clin. Med..

[3] McDonagh T.A., Metra M., Adamo M., Gardner R.S., Baumbach A., Böhm M., Burri H., Butler J., Čelutkienė J., Chioncel O. (2023). 2023 Focused Update of the 2021 ESC Guidelines for the diagnosis and treatment of acute and chronic heart failure. Eur. Heart J..

[4] Tkaczyszyn M., Drozd M., Ponikowski P., Jankowska E.A. (2019). Iron deficiency in heart failure: A 2020 update. Kardiologia Polska.

[5] Jankowska E.A., Rozentryt P., Witkowska A., Nowak J., Hartmann O., Ponikowska B., Borodulin-Nadzieja L., Banasiak W., Polonski L., Filippatos G. (2010). Iron deficiency: An ominous sign in patients with systolic chronic heart failure. Eur. Heart J..

[6] Martens P., Nijst P., Verbrugge F.H., Smeets K., Dupont M., Mullens W. (2018). Impact of iron deficiency on exercise capacity and outcome in heart failure with reduced, mid-range and preserved ejection fraction. Acta Cardiol..

[7] Anker S.D., Comin Colet J., Filippatos G., Willenheimer R., Dickstein K., Drexler H., Lüscher T.F., Bart B., Banasiak W., Niegowska J. (2009). Ferric Carboxymaltose in Patients with Heart Failure and Iron Deficiency. N. Engl. J. Med..

[8] Ponikowski P., Van Veldhuisen D.J., Comin-Colet J., Ertl G., Komajda M., Mareev V., McDonagh T., Parkhomenko A., Tavazzi L., Levesque V. (2015). Beneficial effects of long-term intravenous iron therapy with ferric carboxymaltose in patients with symptomatic heart failure and iron deficiency^†^. Eur. Heart J..

[9] Anker S.D., Kirwan B., van Veldhuisen D.J., Filippatos G., Comin-Colet J., Ruschitzka F., Lüscher T.F., Arutyunov G.P., Motro M., Mori C. (2018). Effects of ferric carboxymaltose on hospitalisations and mortality rates in iron-deficient heart failure patients: An individual patient data meta-analysis. Eur. J. Heart Fail..

[10] Campodonico J., Nicoli F., Motta I., De Amicis M.M., Bonomi A., Cappellini M., Agostoni P. (2021). Prognostic role of transferrin saturation in heart failure patients. Eur. J. Prev. Cardiol..

[11] Grote Beverborg N., Klip I.T., Meijers W.C., Voors A.A., Vegter E.L., van der Wal H.H., Swinkels D.W., van Pelt J., Mulder A.B., Bulstra S.K. (2018). Definition of Iron Deficiency Based on the Gold Standard of Bone Marrow Iron Staining in Heart Failure Patients. Circ. Heart Fail..

[12] Wish J.B. (2006). Assessing iron status: Beyond serum ferritin and transferrin saturation. Clin. J. Am. Soc. Nephrol..

[13] Suominen P., Punnonen K., Rajamäki A., Irjala K. (1998). Serum transferrin receptor and transferrin receptor-ferritin index identify healthy subjects with subclinical iron deficits. Blood.

[14] Moliner P., Jankowska E.A., van Veldhuisen D.J., Farre N., Rozentryt P., Enjuanes C., Polonski L., Meroño O., Voors A.A., Ponikowski P. (2017). Clinical correlates and prognostic impact of impaired iron storage versus impaired iron transport in an international cohort of 1821 patients with chronic heart failure. Int. J. Cardiol..

[15] Sierpinski R., Josiak K., Suchocki T., Wojtas-Polc K., Mazur G., Butrym A., Rozentryt P., van der Meer P., Comin-Colet J., von Haehling S. (2021). High soluble transferrin receptor in patients with heart failure: A measure of iron deficiency and a strong predictor of mortality. Eur. J. Heart Fail..

[16] Ras-Jiménez M.D.M., Ramos-Polo R., Francesch Manzano J., Corbella Santano M., Morillas Climent H., Jose-Bazán N., Jiménez-Marrero S., Garcimartin Cerezo P., Yun Viladomat S., Moliner Borja P. (2023). Soluble Transferrin Receptor as Iron Deficiency Biomarker: Impact on Exercise Capacity in Heart Failure Patients. J. Pers. Med..

[17] Jankowska E.A., Kasztura M., Sokolski M., Bronisz M., Nawrocka S., Kowska-Florek W.O., Ski R.Z., Biegus J., Owski P.S., Banasiak W. (2014). Iron deficiency defined as depleted iron stores accompanied by unmet cellular iron requirements identifies patients at the highest risk of death after an episode of acute heart failure. Eur. Heart J..

[18] Restrepo-Gallego M., Díaz L.E., Rondó P.H. (2021). Classic and emergent indicators for the assessment of human iron status. Crit. Rev. Food Sci. Nutr..

[19] Leszek P., Sochanowicz B., Szperl M., Kolsut P., Brzóska K., Piotrowski W., Rywik T.M., Danko B., Polkowska-Motrenko H., Różański J.M. (2012). Myocardial iron homeostasis in advanced chronic heart failure patients. Int. J. Cardiol..

[20] Zhu S., Liu C., Zhao C., Chen G., Meng S., Hong M., Xiang M., Xie Y. (2022). Increased Serum Soluble Transferrin Receptor Levels Were Associated With High Prevalence of Cardiovascular Diseases: Insights From the National Health and Nutrition Examination Survey 2017–2018. Front. Cell Dev. Biol..

[21] Fernández-Real J.M., Moreno J.M., López-Bermejo A., Chico B., Vendrell J., Ricart W., Perseghin G., Lattuada G., De Cobelli F., Ragogna F. (2007). Circulating soluble transferrin receptor according to glucose tolerance status and insulin sensitivity. Diabetes Care.

