# Supplementary material for: Prognostic Role of Tissue Iron Deficiency Measured by sTfR Levels in Heart Failure Patients without Systemic Iron Deficiency or Anemia
Source: J Clin Med. 2024 Aug 13;13(16):4742. doi: 10.3390/jcm13164742 (PMC11355225; doi:10.3390/jcm13164742)
Supplement: Supplementary file 1 [file jcm-13-04742-s001.zip › jcm-3110256-supplementary.pdf]

Supplementary Table S1. Sex, age and LVEF adjusted GAM models to explore the parametric and non-parametric associations between sTfR and biomarkers indicating cardiac damage (troponin and NT-proBNP), renin-angiotensin-aldosterone system activation (aldosterone, serum ACE activity and plasma renin activity), inflammatory status (C-reactive protein) and cellular response to tissular hypoxia (endogenous erythropoietin).

	Log (sTfR)	
**Dependent variables**	**Parametric *p*-value**	**Non-parametric *p*-value**
Troponin	0.0842	0.16634
NT-proBNP	0.02181	0.2829
Aldosterone	0.19243	0.05924
Serum ACE activity	0.62026	0.4636
Plasma renin activity	0.88537	0.49952
C-reactive protein	0.001117	0.01478
Albumin	0.003473	0.06326
Endogenous erythropoietin	0.003769	0.001205

PANEL A. Cardiac biomarkers

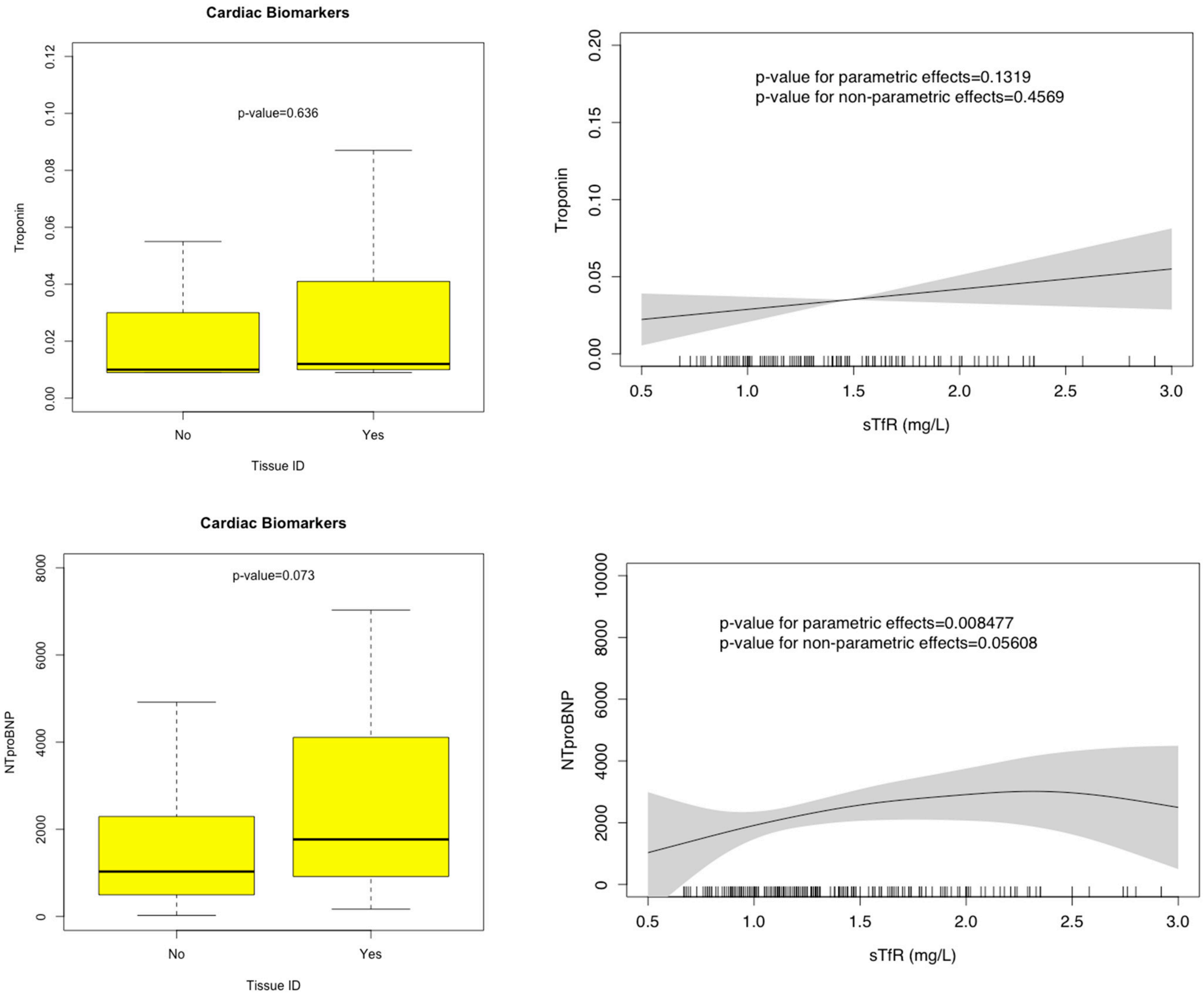

PANEL B. Renin-Angiotensin-Aldosterone system activation biomarkers

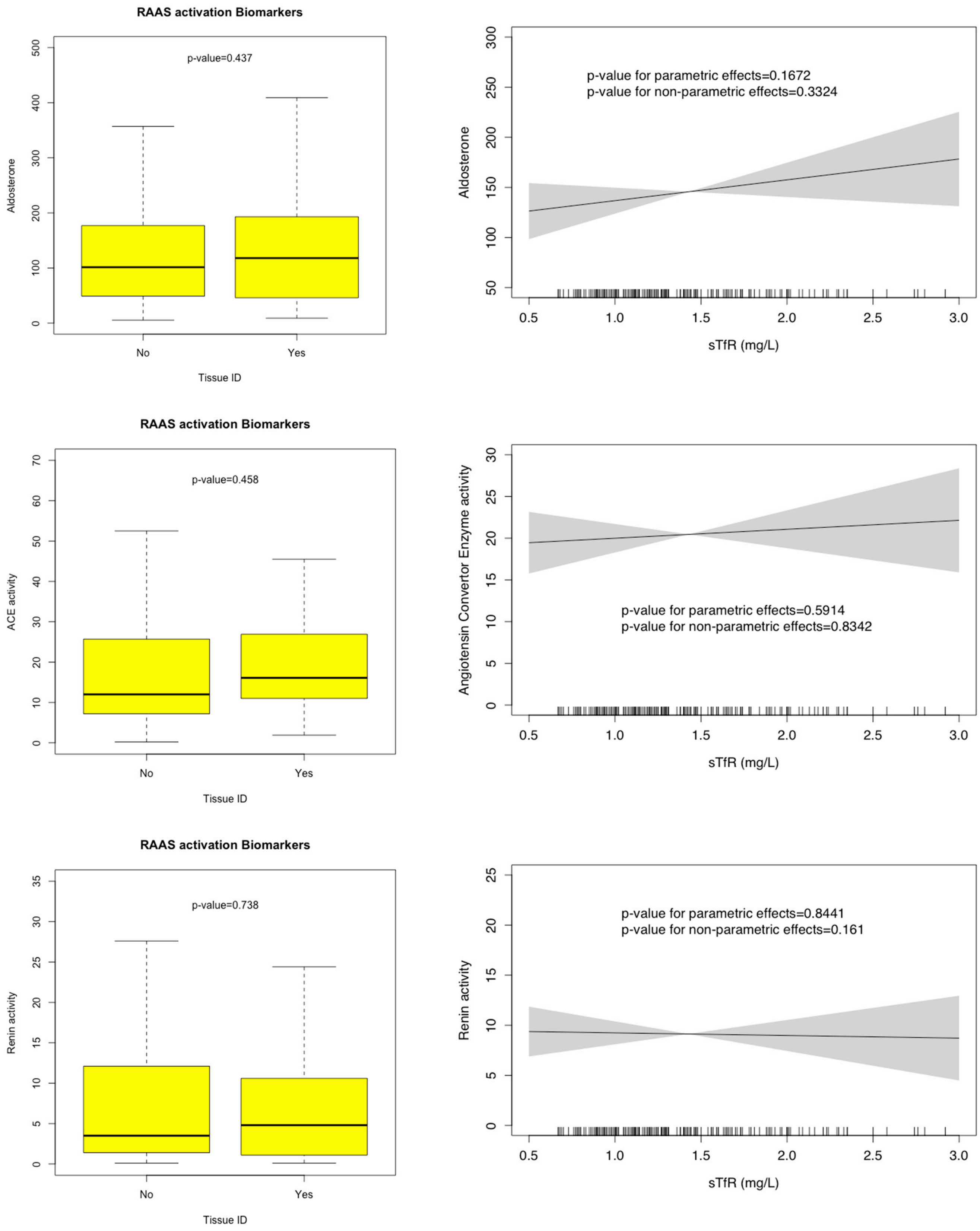

PANEL C. Cellular response to hypoxia biomarkers

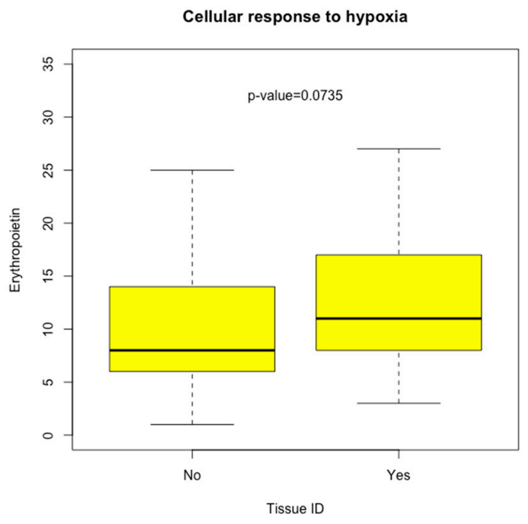

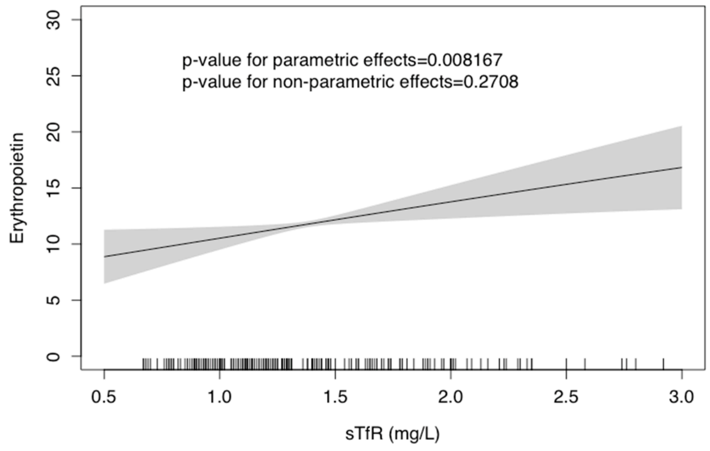

PANEL D. Inflammatory status biomarkers

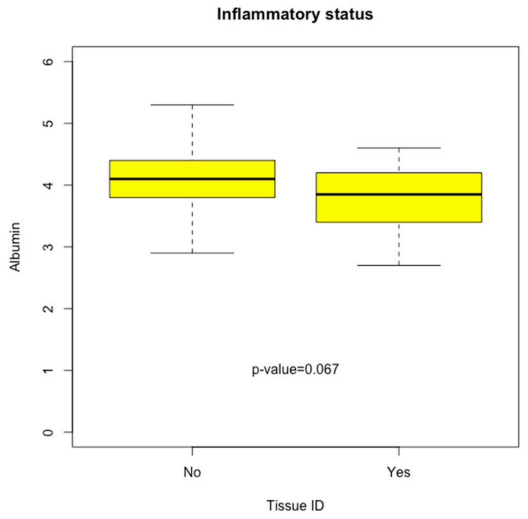

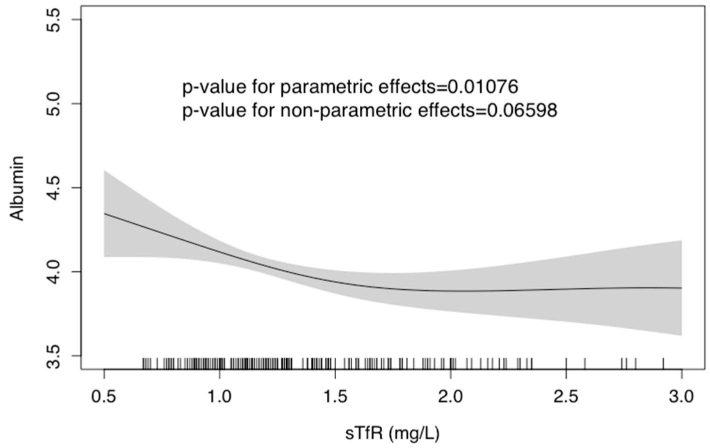

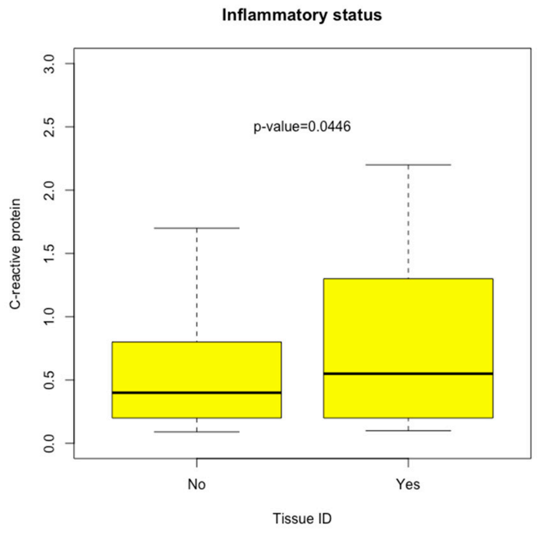

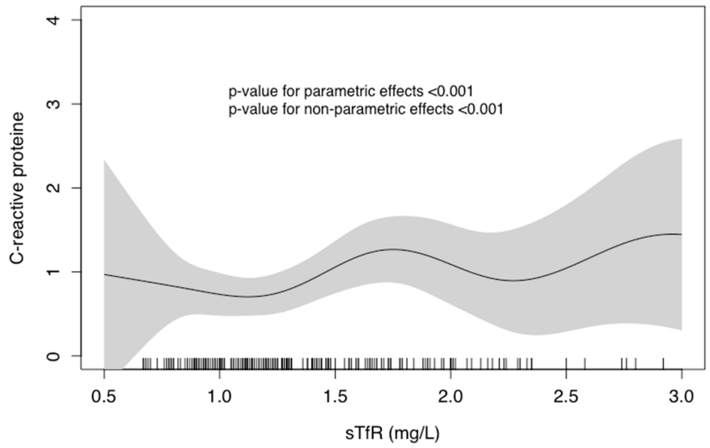

Supplementary Figure S1. Boxplots (showing the mean and standard deviation) of biomarkers according to the presence (Tissue ID [+]) or absence (Tissue ID [−]) and Univariate Generalized Additive Models (GAM) exploring the associations between sTfR levels and the biomarkers that indicate cardiac damage (troponin and NT-proBNP), renin-angiotensin-aldosterone system activation (aldosterone, serum ACE activity and plasma renin

activity), inflammatory status (C-reactive protein) and cellular response to tissular hypoxia (endogenous erythropoietin) tissue ID. (**A**) Cardiac biomarkers. (**B**) Renin-Angiotensin-Aldosterone system activation biomarkers. (**C**) Cellular response to hypoxia biomarkers. (**D**) Inflammatory status biomarkers.
